# Social Networks May Shape Visually Impaired Older Adults’ Occupational Engagement: A Narrative Inquiry

**DOI:** 10.1177/15394492221078315

**Published:** 2022-02-22

**Authors:** Ji Won Kang, Colleen McGrath, Debbie Laliberte Rudman, Carri Hand

**Affiliations:** 1 University of Waterloo, Ontario, Canada; 2 Western University, London, Ontario, Canada

**Keywords:** occupational science, rehabilitation, visual perception, social participation, older adults

## Abstract

Age-related vision loss (ARVL) has been shown to interfere with older adults’ occupational engagement. The primary purpose was to examine the role social networks play in facilitating/constraining engagement in desired occupations for older adults with ARVL. This study adopted a constructivist narrative methodology. Five older adults, ≥ 60 years of age with ARVL, participated in three virtual interviews, which were coded using thematic analysis. Three overarching themes were identified: (a) Diverse Social Networks Fulfill Different Occupational and Psychosocial Needs, (b) Retaining a Sense of Independence through Seeking Reciprocity in Social Relationships, and (c) Community Mobility and Technology Support as Essential for Preserving Social Relationships. Findings broaden understandings of how informal/formal social networks are involved in shaping visually-impaired older adults’ adaptation to ARVL and related occupational changes. Findings may help improve the quality and delivery of low-vision rehabilitation services to optimize their contribution to occupational engagement.

## Introduction

According to the World Health Organization (WHO, 2021), age-related vision loss (ARVL) is the third leading cause of vision impairment globally, and with the aging population, the number of older adults with uncorrectable vision impairment is projected to increase two-fold by 2050. ARVL, a term that includes macular degeneration, glaucoma, and diabetic retinopathy, refers to a permanent visual impairment that is non-correctable to normal visual acuity with standard optical devices or surgery (Watson, 2001).

Age-related vision loss impacts the ability of older adults to engage in meaningful occupation, a term that entails both the performance of an activity and the subjective meanings, emotions, and significance associated with the action (Hocking, 2009). Previous research demonstrates the disruptive impact of ARVL on leisure, instrumental activities of daily living (IADLs), activities of daily living (ADLs), and social interactions (Berger & Porell, 2008; Girdler et al., 2008). Such limitations to older adults’ occupational engagement can contribute to higher incidence of mental illness such as anxiety, depression, and other mood disorders. In addition, adverse physical and social outcomes, such as higher fall-frequencies, social isolation, and withdrawal from social roles and relationships, have been connected to these disruptions to occupational engagement (Berger & Porell, 2008; Girdler et al., 2008).

Social networks are important mediators of individuals’ occupational engagement that can shape people’s awareness, and access to, various resources, which can, in turn, determine their access to opportunities, attitudes, and ability to act upon an occupation (Berkman et al., 2000). Social networks provide various types of social support to older adults, including emotional (defined as support based on trust, empathy, compassion, and affection), informational (defined as information and advice that are useful for problem-solving and decision making), instrumental (defined as tangible or materialistic goods and services), and positive social interaction (defined as sense of companionship or having others to do activities with) (Berkman et al., 2000; Sherbourne & Stewart, 1991).

To date, existing research regarding social networks and ARVL has primarily focused on the impact social networks have on the emotional and psychosocial wellbeing of individuals with ARVL (Papadopoulos et al., 2014; Reinhardt, 2001), as well as the challenges ARVL imposes on maintaining existing, or building new, social networks (Wang & Boerner, 2008). The link between visually impaired older adults’ social networks and occupational engagement has been less researched, with a few notable exceptions (Kaldenberg, 2019; McGrath & Astell, 2016). These studies have focused on visually impaired older adults’ ADLs, IADLs, as well as some limited aspects of social life, including interpersonal interactions and community/civic engagement. However, the studies have failed to address how social networks are involved in broader domains of occupations that older adults with ARVL engage in. In addition, while the above studies depict a strong relationship between the size of the social network, quality of network relationships, and improved occupational engagement among visually impaired older adults, and thereby, demonstrates the potential significance of social support for occupations, the mechanisms behind how social networks shape older adult’s occupational engagement remains unclear. Thus, the objective of this narrative study was to uncover how older adults with ARVL story and make meaning of the roles that their social networks play in both facilitating and constraining their engagement in desired occupations.

The current study can distinguish how different social networks contribute to various occupations of visually impaired older adults. This has practical implications for developing future vision-care policies, training for technology usage, leisure programs, and community mobility interventions for older adults living with visual disabilities.

## Methodology and Methods

This study adopted a constructivist paradigmatic approach to Reissman’s narrative inquiry methodology. Narrative inquiry aims to interpret how people convey their experiences and make sense of their realities through storytelling, and hence captures holistic stories as primary source of data (Reissman, 2008). The study was grounded theoretically in social capital theory (Burt, 2001). Social capital theory suggests that social networks influence how people fulfill their goals and adapt to challenges in their lives, by providing them with different types, quality, and quantity of resources. Within social capital theory, resources that flow within homogeneous social networks (intra-group members) are often classified as bonding forms of social capital, whereas resources exchanged through interactions with heterogeneous social networks (inter-group members) are referred to as bridging social capital (Burt, 2001). The present study analyzes how bonding and bridging social capital, provided by social networks, are used separately to support distinct occupations and how they are used in combination with one another to augment engagement in an occupational domain.

### Participant Recruitment and Inclusion Criteria

The study took place in London, ON, Canada. Enrolled participants met the following inclusion criteria: (a) aged 60 years and older, (b) living with ARVL (including macular degeneration, glaucoma, and/or diabetic retinopathy), (c) have lived with ARVL for at least 6 months, (d) able to communicate in conversational English, and (e) not have any cognitive or (non-corrected) hearing impairments that would interfere with data collection. Five participants were recruited through convenience sampling from a vision rehabilitation service provider and an education center for retired older adults. This sample size was decided based on an evidence-based approach after reviewing several narrative studies that reflected the guiding principles of this methodology (i.e., promotion of in-depth case-oriented inquiry) (Butina, 2015; James, 2018; Patton, 2002). Researchers above, achieved richness in their data by conducting multiple interviews with each participant (and building upon previous responses) rather than using larger sample sizes. In the above studies, sample sizes ranged from 1 to 10 participants, and all researchers concluded that they reached data saturation, where no new themes or codes arose from further sampling.

### Data Collection

The study received approval from the Western University Institutional Review Board (reference number: 115681). Data were collected over a period of 7 months, during which there was an outbreak of COVID-19 (an infectious disease caused by SARS-COV-2 novel coronavirus that started in 2019). Data collection included three virtual, semi-structured interviews via Zoom or phone calls, wherein four participants engaged in telephone interviews, and one participant engaged via Zoom. The severity of vision loss was defined using self-reported measures, where participants rated their vision impairment as mild, intermediate, or severe (see Table 1 for a detailed description of the Data Collection Protocol). Before the first interview, participants provided informed consent (either written or verbal), while process consent was obtained prior to subsequent interview sessions. All interviews were audio recorded, transcribed verbatim, and conducted by the first author.

**Table 1.** Data Collection Protocol.

Interview 1: Narrative interview	Interview 2: Semi-structured interview	Interview 3: Feedback interview
Based on Wengraf’s (2001) Biographic-Narrative-Interpretive Method (BNIM) interviewing approach, participants were asked to respond to two open-ended questions regarding their experiences of interacting with social networks and engaging in desired occupations while living with ARVL: (1) “Can you tell me about the types of activities you participate in since being diagnosed with vision loss? How has your participation in those activities changed since experiencing vision loss?”; and (2) “Can you tell me about a time when someone either helped or hindered your participation in those activities?” Follow-up questions were asked only after participants finished telling their story. Participants’ demographic data were collected at the end of the interview (see Table 2 for the demographic profiles of participants). The interview length was approximately 1 hr.	Based on Wengraf’s (2001) BNIM approach, the researcher prepared a set of semi-structured, in-depth follow-up questions, based on the responses generated from the first interview. These open-ended questions were asked to participants, following Patton (2002)’s general interview guide approach. Example questions asked during Interview two included: (1) “You’ve mentioned that your vision loss has been a gradual process. Can you talk more about how that gradual process has impacted your activities and your level of reliance on your social networks? Do you think your experience is different from someone that may have experienced a more sudden loss of vision?”; and (2) “During our first interview, you talked about how you and your daughters have glaucoma. Given that you are all experiencing the same diagnosis, what type of social support do they provide to you? What type of support do you provide to them?” The interview length was approximately 1 hour. Participants’ narratives were rendered based on the data they shared from the first and second interviews.	Participants were invited to reflect and provide feedback on their narrative accounts, which were re-constructed by the researcher and emailed to the participants prior to the third interview session. These sessions were no more than 10-15 min long. Examples of requested changes included minor grammatical edits, removal of one quote, and adding information about single motherhood to one participant’s parenting experience. The feedback was integrated into the analysis process and used to alter the final rendering of participants’ narratives.

*Note.* BNIM = biographic-narrative-interpretive method; ARVL = age-related vision loss.

### Data Analysis

Data were analyzed through three iterative steps, including coding the transcripts using thematic analysis, reconstruction of participant narratives, and generation of overarching themes between, and across, all participants’ data sets. During the thematic analysis stage, the primary researcher engaged in line-by-line coding of each transcript by using both inductive, open-ended coding and abductive coding informed by social capital theory, focusing on the content of participants’ experiences (Fraser, 2004; Reissman, 2008). In the reconstruction process, each narrative was organized by storylines, under which the major plot points were loosely ordered chronologically. Participant quotes were then inserted back into the narratives at relevant places (Nasheeda et al., 2019; Wengraf, 2001). Finally, the first and second author separately generated overarching themes across all participants by cross-comparing the codes, storylines, and reconstructed narratives specific to each participant (Fraser, 2004), and arrived at three overarching themes.

## Results

Three themes that address how social networks facilitate as well as constrain engagement in occupation for older adults with ARVL are discussed, namely: *(a) Diverse Social Networks Fulfill Different Occupational and Psychosocial Needs, (b) Retaining a Sense of Independence through Seeking Reciprocity in Social Relationships, and (c) Community Mobility and Technology Support as Essential for Preserving Social Relationships*. To optimize anonymity, the names of people, organizations, and places were removed from the quotes integrated into the findings, and assigned pseudonyms were used. As shown in Table 2, participants were diverse in relation to several demographic characteristics, including age, income, number of children, and degree of vision loss, but all were female, Caucasian, and had some form of post-secondary education.

**Table 2.** Demographic Profiles of Participants.

Participant’s pseudonym	Linda	Brendell	Jenny	Joanna	Grace
Age	68	91	67	85	82
Gender	Female	Female	Female	Female	Female
Racial/Ethnic Group	Caucasian	Caucasian	Caucasian	Caucasian	Caucasian
Level of Education Completed	Graduate degree	Completed university	Completed university	Completed university	Some college
Annual Household Income	$50,000–$100,000	> $100,000	$50,000–$100,000	$50,000–$100,000	<$25,000
ARVL Diagnosis	Glaucoma	Age-related macular degeneration	Glaucoma	Age-related macular degeneration	Age-related macular degeneration
Degree of Vision Loss	Mild (Partially sighted)	Mild (Partially sighted)	Intermediate (Partially sighted)	Severe (Partially sighted)	Severe (Partially sighted)
Time since diagnosis of ARVL	15 years	20 years	10 years	20 years	25 years
Marital Status	Divorced	Widowed	Married	Widowed	Widowed
Number of Children	2	1	2	2	4

*Note.* ARVL = age-related vision loss.

### Diverse Social Networks Fulfill Different Occupational and Psychosocial Needs

Participants discussed various difficulties they faced at different stages of vision loss, including accomplishing IADLs (i.e., cooking, financial management, computer use, reading, grocery shopping), occupations involving group interactions, community mobility (i.e., attending medical appointments), and leisure occupations (i.e., camping, watching TV). At the same time, participants described how their social networks helped to compensate for the occupational losses associated with their ARVL. In particular, participants’ formal social networks, defined as networks based in transactional connections where the services/supports exchanged between members are paid for (i.e., paid workers, health care professionals, non-governmental organizations), provided both instrumental and informational support. Table 3 summarizes some of the social supports provided by various formal network members and demonstrates how they facilitated participants’ engagement in diverse occupations.

**Table 3.** Instrumental and Informational Support Provided by Formal Networks.

Formal network members	Social support	Sample participant quotes	Relations to occupational engagement
Healthcare Professionals (i.e., optometrist and ophthalmologist)	Provide access to healthcare referrals, diagnostic tests, medications, surgery, and information regarding their progression of vision loss	*“Nowadays the optometrists, they always check the pressure in your eye. As soon as they detect an increase in your intraocular pressure, they monitor that, and if it reaches a certain critical point, they send you to see the ophthalmologist. When you reach a point that the high pressure doesn’t respond anymore to the medications, the next step is usually laser treatment. So, they have a number of techniques and medications that help to slow down the progress of your vision loss, and I need those checkups so I can still drive, watch TV, recognize faces of people I’m talking to, and do all the other things I’ve done before as long as possible. (Linda)* *“He’s [ophthalmologist] done surgery on my eye for glaucoma, and with the surgery, I don’t have to use the drops for glaucoma anymore. Before, I was using the drops, but it was getting really bad, it felt like I was looking through water. And I didn’t want my vision to interfere with my life even more. So I got the surgery.” (Jenny)*	Providing medical services that prevent further vision loss helps prolong participants’ engagement in occupations that rely heavily on vision such as driving and comprehending non-verbal communication cues (i.e., body language, facial expressions, etc.).
Low-vision rehabilitation organizations	Provide access to low-vision assistive technologies (i.e., digital hand-held video magnifier, CCTV desktop magnifier, text-to-speech software, iPad, etc.), phone operator services, and home visits for orientation and mobility instruction	*“I have a CCTV that I got through the [name of low-vision rehabilitation organization], and I can fill out forms using it. It’s getting difficult [to fill out forms] because of my vision loss. I have trouble finding out just where that mark should be or where the signing place should be . . . I used to know a lady that worked for [name of low vision rehabilitation organization], who would come and put little bumps on equipment so that you would know where the on and off switches were. I need that now on my microwave and this new stove. I don’t know how to run the oven yet.” (Grace)* *“I use this magnifying glass, and I use it a lot every day. I take it to grocery shopping if I want to read a label on a can, for example, or price of something on it . . . I use that for doing bank racks or checking the visa too.” (Joanna)*	Low vision rehabilitation services help participants navigate in their homes more safely by creating more vision friendly spaces, facilitating use of their home appliances, enabling engagement in IADLs such as cooking, banking, and reading (i.e., labels on prescription bottles, street signs, and instruction manuals, etc.).

*Note.* CCTV = closed circuit television; IADLs = instrumental activities of daily living.

In comparison to formal networks, participants’ informal networks, defined as networks comprised of primary or proximal members who you exchange unpaid support with (i.e., family members and friends) (Kim et al., 2019), provided more diverse types of social support, including companionship as well as emotional, informational, and instrumental support. In particular, participants relied heavily on their peers (i.e., network members sharing a similar age, disability, gender, or other circumstance), who were perceived as having more tacit understanding and empathy for participants’ needs and experiences (see Table 4, for examples, of the comprehensive range of social supports provided by informal networks).

**Table 4.** Comprehensive Range of Social Supports from Informal Networks.

Support types	Linking informal social support with occupational engagement	Sample participant quotes
Instrumental Support	The provision of instrumental support was overwhelmingly provided by family members more so than friends. Family members (i.e., spouse and children) provided technological assistance and financial support (i.e., for internet, taxi service, gym membership, low-vision assistive devices) as well as help with IADLs such as household chores, grocery shopping, cooking, and providing drives to medical appointments and for running errands.	*“My son in the States is going to pay for my Internet connection. And [daughter] and [son-in-law], who live here helped me with grocery shopping. If I wanted to go to [name of grocery store], they would take me, or they would pick up things for me there. And they usually don’t charge me for the full amount of groceries they buy for me. . . I’m also dependent on my daughters to get to doctor’s appointments. And my youngest daughter, she was the one that put [digital audiobook application], on my iPad for me.” (Grace)* *“My husband helps me a lot with the meals, laundry, and everything else outside and inside our house.” (Jenny)*
Companionship	Friendship networks were central to fostering a sense of companionship. Participants’ friends accompanied them in their participation in leisure activities (i.e., watching TV shows, camping, going out for dinner, concerts) and physical activities (i.e., riding bikes and walking)	*“I have a group of friends, and we go to different theater shows, and we go out for dinner at night. So, they provide me with entertainment. Together, we get to go to different places that my husband and I usually don’t go to, like new restaurants and things like that.” (Jenny)* *“I don’t see most of my friends on a regular basis, but there’s these two that stay close to me. We join for dinners or jazz concerts on Saturday nights. Sometimes, I go on a walk with them outside when the weather’s good.” (Joanna)*
Emotional Support	Informal networks provided emotional support by checking in with participants to see how they were managing with their vision loss, showing reciprocal appreciation of each other’s company, and offering a sense of stability/comfort, safety/trust, and positivity. While both family members and friends served as important sources of emotional support, they each fulfilled different needs. For instance, participants sought more sporadic, short-term emotional relief from their friends, while they expected long-term, enduring emotional support from their family. There was an underlying belief amongst participants that their relationships with family members were more robust and likely to last longer (lifetime) than their ties with friends. Participants also generally interacted with their family at more regular intervals (daily phone calls/texts and weekly video meetings) than with their friends whom they interacted with weekly or monthly. Both family and friends provided encouragement, reassurance, empathy, and affection but in the context of different topics (i.e., vision care and general health check-ups, isolation, end of life preparation, etc.), where more impending/serious issues were discussed with family members first.	*“We talk about everything, I tell him [husband] everything, and he tells me mostly everything. He’s my basic and most go-to emotional support person. We always confide in each other. We don’t always see eye to eye, but we understand each other’s point of view. That’s very important . . . My girlfriends and I, for twelve years, we’ve been sticking together doing social things and to talk about our problems, and the reasons for why certain things are happening in life. So, it’s a stress relief. Obviously, they have their own things, I don’t expect them to be there for me all the time, it’s not possible, but it’s just fun to meet them every couple of weeks, for a few hours.” (Jenny)* *“My daughter’s pretty good emotionally, she keeps in touch over the phone and on the Internet every couple of days. And then, she comes to visit every couple of months for a week. If I reach out, she’s there, always and forever, you know? . . . And my brother lives in the same city as me and he lives close. So that’s a bit of a comfort. I know if I’m in any trouble, I got him to contact, he’s sure there for me, too.” (Joanna)*
Advocacy Support	Both family and friends advocated for participants in various social settings so they could participate fully in their desired occupations. For instance, family members advocated for participants during medical appointments by explaining the challenges faced with measuring medication. Friends also advocated for participants in marketplace interactions, protecting them from people trying to take advantage because of their vision loss (i.e., buyers shortchanging participants when they are working as store personnel).	*“[Participant’s daughter] was there [hospital] with me, and she explained to him that I wouldn’t be able to see a measure [on a dosing syringe; for measuring the exact dose of prescribed liquid or cream medicine] . . . When I was in the [Coffee Club], one lady put her cup on the top of her head, saying that she wanted another cup of coffee. And there were a lot of people who understood that I couldn’t see the cup on top of her head. They told her that she should not do that anymore when I was on the job.” (Grace)* *“I play Bridge cards casually with my friends in our neighbourhood. They’ll tell me if the cards aren’t right in my hands, and I need to see. Like, I can ask, ‘what’s that’? And they’ll tell me. And if other people from our complex join in, my friends will let them know that I can’t see well, and explain why we’re using larger cards.” (Joanna)*
Peer Support—ARVL-Specific Informational and Emotional Support	Within family and friendship supports, peer support seemed particularly important for ARVL-specific adaptation. For instance, participants relied on their peers the most for gathering information relating to vision care (i.e., information about the progression of their vision loss, medications, and strategies they have used to overcome vision challenges) and emotional validation/reassurance when coping with occupational restrictions caused by vision loss. Peer networks also reduced the following barriers to LVRS accessibility: (1) a lack of awareness of available resources due to limited/discontinued communication between service providers and service users; and (2) the physical inconvenience of having to travel to far-away locations to access services. For instance, peers who had personal ties with low vision rehabilitation organizations informed participants of where they accessed specific assistive devices (i.e., braille products, canes, magnifiers, lighting, etc.) and provided transportations to related shops, bridging the severed ties between participants and rehab organizations. By improving access to LVRS, peer networks further contributed to augmenting participants’ ability to engage in occupation.	*“And so, I find that being in that organization, that is all senior people, that there is a lot of understanding and a lot of patience with people needing that extra time or the extra help or not being able to see something on the board.” (Linda)* *“Having both my daughters have glaucoma, if I say something wrong about glaucoma, they’ll correct me. The older one gives me more information than anybody else does about it. Like I’ll do things, and she goes like, ‘oh you shouldn’t do that, it’s not good for glaucoma.’” (Jenny)* *“It would be nice to know when new things come out. I don’t have any communication with them [name of low vision rehabilitation organization]. I have a little catalogue of theirs, but honestly, I’m not sure what they can do . . . I have a friend who was there for her own reasons this week, and they told her about the shop where she can get different magnifying glasses and things. But I can’t drive there by myself, so I’m going to go there with her next week. But I’ve had very limited access to them, and usually, I only realize something new has come out when my friend tells me about it.” (Joanna)*

*Note.* LVRS = low vision rehabilitation services; IADLs = instrumental activities of daily living; ARVL = age-related vision loss.

### Retaining a Sense of Independence Through Seeking Reciprocity in Social Relationships

Analysis indicated that maintaining independence was an important part of how participants maintained wellbeing while living with ARVL. Participants equated being dependent on others as being a burden and only requested help in social situations where they perceived an imminent risk to their safety and/or for help accessing basic necessities such as food or medical care:

*While shopping for groceries, I often ask where this is, or that is because I can’t find some things. But I try not to be a nuisance to people unless I really need it. (Joanna)*

*I always hesitate [to ask for help], but around my surgeries, I’ve needed my good friends near me . . . Because right after the surgery, you can’t see with that eye for a day or so. With the bad eye, I wouldn’t be able to function. (Linda)*

Here, participants discussed how reciprocal social relationships, based on mutual exchanges of social support, helped them maintain their sense of independence and feel equal to their network members, even while seeking help. As such, reciprocity was crucial for participants when seeking support for desired and necessary occupations:

*It’s not so much that I need, and they [participant’s friends] don’t. So you feel a little freer to ask for help from them if you needed it, whether it be asking for a ride or reading some label, because it’s reciprocal. (Joanna)*

Participants’ ability to reciprocate was influenced by several factors including their financial stability, level of intimacy in relationships, visual function, and perceived similarity in resources possessed by network members and oneself. For instance, growing intimacy in relationships increased the number of ways participants could provide support to others (i.e., acting as a caregiver, assuming the role of confidant, or exchanging emotional support) as they did not have to replicate the exact type of support they received. Having extra income also allowed participants to compensate for their social networks’ time and emotional support with financial support:

*I think it’s therapeutic for her [participant’s neighbour] to talk to me because when she comes, I hear everything she’s done all day. So I’m sure it’s helpful for her as well as me . . . I give my daughter 350 dollars a month because she has very little income. She’s on a disability, and that lets her stay in the apartment she’s in. (Brendell)*

In addition, possessing similar resources as others (in terms of health status, age, income, etc.) and having greater visual function were perceived by participants as giving them greater ability to help others, and participants reported finding it easier to be of help to their peers with similar disabilities:

*My friends are around my age and dealing with similar things as me; they are not working, some of them have vision loss or arthritis, so I can help them out when they need it . . . It’s harder to help my children because I can’t help them with the food preparation anymore, or other house chores or technology-related things because they all do it better than I ever can with this vision. (Grace)*

### Community Mobility and Technology Support as Essential for Preserving Social Relationships

Social networks were essential in preserving participants’ community engagement as they provided support for community mobility and technology use, which were mediators of social participation that were challenged by ARVL.

ARVL negatively influenced participants’ ability to drive as well as limited their opportunities to access other forms of community mobility, which is defined as “planning and moving around in the community using public or private transportation” (American Occupational Therapy Association, 2008, p. 631). However, participants relied on both their close friends and family members for rides to various leisure and social activities (i.e., attending music concerts, theaters, restaurants, and camping) and sought for drives predominantly from their family members for essential services and/or private errands (i.e., medical appointments, grocery shopping, and trips to the pharmacy):

*My daughter helps me get to my doctor’s appointment and takes me to the pharmacy so I can pick up my prescriptions, and she often does the grocery shopping for me, or brings me along in her grocery trips. (Grace)*

*It used to be, my girlfriends and I usually take turns [driving one another], but now, they’ve [friends] got to take all the turns when we go out to watch ballet performances, visit new restaurant, and other things . . . My husband does all the driving when we’re out camping. (Jenny)*

Assistance from family and friends was also necessary to support participants’ desire to visit family who lived outside of the city as well as to travel to/from volunteer positions and workplaces. As such, support from social networks were closely intertwined with preserving meaningful social relationships and continued participation in productive occupations:

*My daughter’s family lives in [Quebec]. So, until now, I’ve been able to see them quite often because I drive, but soon, I’ll have to rely on taking the train, or asking them [family] to come over if I lose my vision more. (Linda)*

*If I couldn’t drive there [name of volunteer organization], I’d have to ask my colleagues for carpool or resign and that would be a problem. (Brendell)*

In addition to relying on their informal social networks for rides, participants also accessed public transit (i.e., buses and trains) and/or private transportation services (i.e., taxis). Here, formal support networks such as low-vision rehabilitation services enabled participants to maintain their community mobility using public transportation, by providing subsidized transit fares for people with registered vision loss:

*I have a [name of low-vision rehabilitation organization] card and transit that says that I can ride any of the city buses. It costs me ten dollars per year. They can’t get any cheaper. (Grace)*

Along with community mobility, social networks enabled participants’ use of technology which was an essential support to participants’ social participation during the time of data collection, in which restrictions related to the COVID-19 pandemic interfered with many social occupations that involved physical interaction with others. Participants discussed using virtual means of social connection (i.e., phone, Zoom, email, and Facebook), as opposed to in-person, to support such occupations as attending health care appointments, volunteering, engaging in leisure activities, and interacting with family and friends:

*During the COVID-19, we’ve [name of volunteer organization] had to shut down. So, since March, we haven’t been able to do any of that [executive meetings, peer-based courses, and programs, etc.]. But we’ve started having zoom meetings, and we’re preparing to launch all of our forces in the fall via zoom. (Linda)*

*I don’t know if my vision has changed, but yes, I don’t see my doctor. It’s [medical appointment] mostly by phone if there’s anything wrong. It’s quite different, and I’d much rather be there in person but he’s very good on the phone. (Joanna)*

The ability to use online technologies, however, was complicated by the functional difficulties associated with their ARVL. For instance, many participants experienced fatigue and eye strain when working on computer monitors, thereby limiting the amount of time they could play online games or hold online meetings. Others expressed that many of the technologies they use daily (i.e., Facebook, audiobook apps, Zoom, and speech-to-text programs) do not have an intuitive interface for older, visually impaired users (i.e., making ineffective use of visual clues to display web content and presenting text or other important information as an image which may not be read by a screen reader):

*On the [Seron Scrabble Club], we’re all playing online instead of going to the meeting where we usually would play each other in person. So we’re using online capabilities to do that, and it’s a little bit harder when you can’t see that well, to play for two, three hours on the screen. (Linda)*

*It takes me long to browse for things on the internet even when I’m using my screen reader because it reads every word on the page. Sometimes, they have poor descriptions of pictures, like saying it’s a* ‘*photo*’ *. . . I rely a lot on colors and bigger fonts to peruse the page, but some site designs make it difficult to navigate the content. (Joanna)*

Here, participants opted to ask their informal networks to set up or help them learn how to operate these technologies as well as received structured group training from their formal networks (i.e., retirement home staff). For instance, participants learned how to make use of Zoom control panel and Facebook action shortcuts compatible with their screen readers to navigate the site more easily:

*[Fox Hollow; retirement home] staff is trying to get things working again and people to interact with each other [during COVID-19]. They are going to help with Zoom meetings and Facebook interaction . . . They said Facebook has keyboard shortcuts that are compatible with most screen readers, so I going to ask them to teach me how to use those. (Grace)*

*She’s [participant’s neighbour] very good with mechanical things. She set the whole program [Zoom] up for me and when I had to host a meeting, she helped me use the control panel, like sharing my screen and inviting people. (Brendell)*

### Discussion

The present study investigated how social networks both facilitate and constrain engagement in occupation for older adults with ARVL. Findings of this study expand our existing knowledge regarding the support provided by different social network members. It also demonstrates how these support types work separately, or in conjunction, with one another to facilitate the engagement of visually impaired older adults in diverse domains of occupation. Formal networks provided mainly informational and instrumental support relating to LVRS or medical services, while informal networks provided companionship, emotional, informational, instrumental, and peer support. Here, family members and friends offered distinct supports that had unique effects on participants’ occupational engagement. Instrumental support and long, enduring emotional support from family members, facilitated participants’ IADLs within and outside the home. Provision of companionship and short, sporadic emotional support from friendship networks enabled participants’ engagement in social, leisure, and physical occupations. Both family and friendship networks participated in advocacy roles (i.e., asking for accommodations or information on behalf of participants and reducing discriminatory behaviors against vision loss) that supported participant’s engagement in health care, marketplace, and volunteer activities. While McIlvane and Reinhardt (2001) similarly associated family networks with long-term provision of instrumental and emotional support and friendship networks with short-term provision of companionship, they have not linked social support to occupational engagement. In addition, existing social support frameworks rarely address advocacy as a type of support despite its benefits for visually impaired older adults’ occupational engagement, which suggests the need to expand such frameworks for people with ARVL. Furthermore, within informal networks, peer support, based on similar experiential knowledge (i.e., providing in-depth insider information and tacit understanding of shared circumstances), was perceived perhaps most positively by older adults with ARVL and was used to support participation in a variety of occupations. This finding is in line with Kaldenberg’s (2019) and Silverman et al.’s (2017) works which showed that peer networks, who shared a disability with visually impaired older adults, offered unique emotional and informational support beyond those provided from ‘normal’-sighted networks. Previous research, however, did not connect peer support specifically to the occupational engagement of visually impaired older adults. On the other hand, the current study focused on how peer networks connected participants to LVRS, helped them emotionally adapt to the changes in their occupations caused by ARVL, and shared strategies to overcome functional challenges while engaging in various social roles.

The present study highlighted the importance of reciprocal relationships (through mutual exchanges of social support) in supporting the participants’ occupational engagement. In line with Reinhardt’s (2001) cross-sectional study involving older adults with ARVL, the present study demonstrated that reciprocity is more easily achieved in intimate social relationships, as there is increased substitutability of support types exchanged. Other gerontological studies investigating the strategies older adults use to achieve reciprocity (Espvall & Dellgran, 2010; Verbrugge & Chan, 2008) reported how they exchange time for money (i.e., doing household chores, babysitting, advising on family matters, etc.) and vice versa (i.e., giving financial help to children, buying gifts, paying for gas money in return for driving, etc.), which emphasized the importance of economic stability for reciprocity. Although participants in these above-noted studies did not have ARVL, the current study observed similar strategies employed by visually impaired older adults. Along with these factors (financial stability and growing intimacy in relationships), the present study also noted the importance of visual function and perceived similarity in resources possessed by network members (i.e., time, energy, health status, educational attainment, etc.) and oneself for obtaining equal exchanges of reciprocity within social relationships.

The current study also highlighted the importance of social networks in supporting participants’ community mobility and technology use, which were both crucial mediators of participants’ engagement in social occupations. Here, participants planned around their current or potential future driving cessation by using alternative modes of transportation such as public transit and relying on informal networks (i.e., spouse, children, siblings, or friends) for rides. This finding is supported by Schryer’s et al. (2019) research, which examined the driving patterns of older adults with vision impairment and found that those with more advanced vision loss are more likely to exercise driving restrictions (i.e., avoid driving at night, in unfamiliar situations, and for long distances) and preferred to rely on other drivers for rides. However, the current study adds to previous research as it demonstrates that community mobility supports from family members and friends were used for distinct types of occupations, with family members facilitating community mobility to support engagement in essential occupations, such as attending medical appointments, whereas friendship networks were sought to support community mobility needed to access leisure activities. Finally, the study findings were in support of existing literature on the use of assistive technology by visually impaired older adults to support their daily occupations (Fok et al., 2011; McGrath & Astell, 2016). Specifically, Fok et al. (2011) identified that technology use promoted social engagement among visually impaired older adults as they relied on assistive technology to manage their community mobility. In line with Fok et al.’s (2011) research, the current study also highlighted how technology facilitates the social connection of visually impaired older adults. However, the present study goes beyond previous study findings by focusing on the role technology plays in substituting in-person interactions with virtual social occupations, and the challenges participants had with technology to support their occupational needs. It also demonstrated how social networks play an important role in training and enhancing participants’ engagement with online technologies, consequently enabling them to maintain their social connections during a physically disconnected time (i.e., because of COVID-19).

### Future Research and Implications

This study expands the existing narratives of visually impaired older adults on their use of social networks in managing ARVL-specific challenges while adjusting to occupational changes. In addition, this study began to elicit answers for the following questions, which need further investigation in future research: (a) How do peer networks of older adults with ARVL affect their occupational engagement differently from the rest of their family or friendship networks?; (b) What factors affect reciprocity in social relationships, and how do these factors predict/relate to the occupational engagement of visually impaired older adults?; and (c) How do social networks support visually impaired older adults’ use of online technologies for virtual occupations?

This study also presents important implications for low-vision rehabilitation practices. For example, participants reported that improved geographic accessibility to vision care services and more active, effective communication between vision care service providers and users are needed. These barriers have been similarly reported in other studies (Southall & Wittich, 2012; Wittich et al., 2013). In relation to the findings of this study, low-vision rehabilitation services may benefit from incorporating visually impaired older adults’ own informal networks in the care process, to mediate continued contact with low-vision rehabilitation services as well as post-diagnosis communication with vision care professionals. In the present study, informal networks provided transportation services to health care appointments, advocated for older adults’ health care needs, and shared information related to vision care and behavioral/emotional coping strategies with participants. Therefore, informal networks may reduce the physical and informational barriers to accessing vision care services and enable older adults to better express their concerns and occupational needs in health care settings. This may help facilitate more open communication between health care professionals and older adults and improve the quality of vision care received and its capacity to support occupational engagement.

### Limitations

The limitations of this study relate to the small sample size and homogeneity in the participant pool (five well-educated Caucasian women), perhaps resulting from the use of convenience sampling. Such a sampling method was used to minimize the negative impact of COVID-19 on recruitment efforts (i.e., due to social distancing regulations, redeployment of vision rehab staff, and prioritization of COVID-19-centric research) and expedite access to available participants. Considering that this study’s findings are restricted to a narrow social context, future research may benefit from investigating how visually impaired older adults’ social support use and occupational experiences intersect with other axis of difference such as ethno-racial identity, gender, and social class. With more diversity reflected, results may depict expansion in older adults’ patterns of help-seeking behaviors, types of occupations engaged, and barriers faced while engaging in occupations or exchanging social support.

## Conclusion

This narrative study explored the lives of five older adults with ARVL to highlight the complex ways that informal and formal social networks shaped their engagement in meaningful occupation. By demonstrating older adults’ unique patterns of occupational engagement, the current study highlights the benefits of social support for maintaining occupational engagement while living with ARVL. The findings of this study may contribute to future gerontological and occupational science research by helping to expand understanding of how social support networks facilitate the occupational engagement of older adults aging with vision loss.
